# Supplementary material for: Chemical Mapping of Excitons in Halide Double Perovskites
Source: Nano Lett. 2023 Sep 1;23(17):8155–61. doi: 10.1021/acs.nanolett.3c02285 (PMC10510582; doi:10.1021/acs.nanolett.3c02285)
Supplement: Supplementary file 1 — nl3c02285_si_001.pdf [file nl3c02285_si_001.pdf]

# Supporting Information for Chemical Mapping of Excitons in Halide Double Perovskites

Raisa-Ioana Biega,[†] Yinan Chen,[‡] Marina R. Filip,[*,‡] and Linn Leppert[*,†]

†*MESA+ Institute for Nanotechnology, University of Twente, 7500 AE Enschede, The Netherlands*

‡*Department of Physics, University of Oxford, Clarendon Laboratory, Oxford, OX1 3PU, United Kingdom*

E-mail: marina.filip@physics.ox.ac.uk; l.leppert@utwente.nl

## Contents

**1 Methodological and computational details** **S2**

1.1 Electronic structure . . . . . . . . . . . . . . . . . . . . . . . . . . . . . . . . S3

1.2 Optical properties . . . . . . . . . . . . . . . . . . . . . . . . . . . . . . . . . S7

**2 Wannier-Mott model** **S8**

**3 Dielectric screening** **S10**

## List of Tables

S1 Computational settings for electronic structure calculations . . . . . . . . . . . S16

S2 Computational settings for optical properties calculations . . . . . . . . . . . S17

S3 Summary of electronic properties: bands gaps and effective masses . . . . . . S18
S4 Exciton binding energies – from BSE, standard and anisotropy-corrected Wannier-Mott model . . . . . . . . . . . . . . . . . . . . . . . . . . . . . . . . . . . . S19
S5 Variation of exciton binding energies with a uniform dielectric screening . . . S20
S6 Static dielectric constant and Thomas-Fermi wave vector . . . . . . . . . . . S21
S7 Summary of electronic properties: bands gaps and effective masses – for the vacancy-ordered double perovskites . . . . . . . . . . . . . . . . . . . . . . . . S21

## List of Figures

S1 Convergence tests for the static dielectric constant . . . . . . . . . . . . . . . . S22
S2 Convergence tests for the QP band gap . . . . . . . . . . . . . . . . . . . . . S23
S3 Convergence tests for exciton binding energy . . . . . . . . . . . . . . . . . . S24
S4 $G_0W_0$+BSE exciton diagram . . . . . . . . . . . . . . . . . . . . . . . . . . S25
S5 Variation of fundamental QP band gap with respect to the static dielectric constant . . . . . . . . . . . . . . . . . . . . . . . . . . . . . . . . . . . . . . S26
S6 Variation of the $G_0W_0$+BSE exciton binding energy with respect to the static dielectric constant . . . . . . . . . . . . . . . . . . . . . . . . . . . . . . . . . S27
S7 Linear optical absorption spectra . . . . . . . . . . . . . . . . . . . . . . . . S28
S8 DFT and $G_0W_0$ band structures . . . . . . . . . . . . . . . . . . . . . . . . S29
S9 Contributions of the orbital character to the electronic QP band structure . S30
S10 Variation of the head of dielectric matrix in reciprocal space . . . . . . . . . S31
S11 Exciton radial probability density in reciprocal space . . . . . . . . . . . . . S32

# 1 Methodological and computational details

For all calculations on double perovskites, we used structures optimized using density functional theory (DFT) as implemented in the plane-wave code Quantum Espresso[1,2] with

the exchange-correlation functional by Perdew, Burke, and Ernzerhofer (PBE),[3] norm-conserving pseudopotentials, a plane wave energy cutoff of 60 Ry and a $4 \times 4 \times 4$ $\Gamma$-centered $\mathbf{k}$-mesh. For the obtained crystal structures, we used the one-shot $G_0W_0$ approach, and construct the zeroth-order Green's function $G_0$ and screened Coulomb interaction $W_0$ from density functional theory (DFT) eigenvalues and eigenfunctions calculated using the PBE exchange-correlation functional including spin-orbit coupling self-consistently whenever necessary. For our $G_0W_0$+BSE calculations, we used the BerkeleyGW code.[4] We report band gaps converged to within 0.1 eV and exciton binding energies converged to within 5 meV. Spin-orbit coupling (SOC) was taken into account self-consistently for those materials for which it significantly affects the band edges. Details of our calculations (Tables S1 and S2), convergence tests (Figure S1, S2 and S3), and all optimized structures can be found below.

## 1.1 Electronic structure

We computed the quasiparticale (QP) energies using the "one-shot" $G_0W_0$ method,[5] where the zeroth-order one-particle Green's function $G_0$ and the screened Coulomb interaction $W_0$ are constructed from a density functional theory (DFT) eigensystem. At the level of first-order perturbation theory, we obtained the QP energies based on the equation

$$E^{\mathrm{QP}}_{n\mathbf{k}} = E^{\mathrm{DFT}}_{n\mathbf{k}} + Z(E^{\mathrm{DFT}}_{n\mathbf{k}}) \langle \psi_{n\mathbf{k}} | \Sigma(E^{\mathrm{DFT}}_{n\mathbf{k}}) - V_{xc} | \psi_{n\mathbf{k}} \rangle \,, \tag{S1}$$

where $E^{\mathrm{QP}}_{n\mathbf{k}}$ and $E^{\mathrm{DFT}}_{n\mathbf{k}}$ are the QP and DFT energies, respectively, $V_{xc}$ is the DFT exchange-correlation potential and $Z(E^{\mathrm{DFT}}_{nk}) = \left[1 - \frac{\partial \operatorname{Re}(\Sigma)}{\partial \omega}\Big|_{\omega=E^{\mathrm{DFT}}_{nk}}\right]^{-1}$ is the QP renormalization factor.

All DFT calculations were performed using the generalized gradient approximation of Perdew Burke and Erzerhof (PBE),[6] as implemented in the Quantum Espresso software package.[1,2] We used norm-conserving pseudopotentials from the PseudoDojo database,[7,8] with the following atomic configurations: Cs ($5s^2 5p^6 6p^1$), Cl ($3s^2 3p^5$), Ag ($4s^2 4p^6 4d^{10} 5s^1$),

Bi ($5d^{10}6s^26p^3$), In ($4d^{10}5s^25p^1$), Sb ($4d^{10}5s^25p^3$), Na ($4s^22p^63s^1$), K ($3s^23p^64s^1$) and Pb ($6s^25d^{10}6p^2$). Scalar relativistic pseudopotentials were used for **Ag/In** and **Na/In** double perovskites, and fully-relativistic pseudopotentials for all other materials. We optimized the geometry of all systems starting from room-temperature experimental X-ray crystal structures and using the PBEsol exchange-correlation functional,[9] a cutoff energy of 60 Ry and a convergence criterion of $0.5 \cdot 10^{-5}$ eV/Ang for the forces and $10^{-8}$ Ry for total energy, respectively and $4 \times 4 \times 4$ **k**-point grids. The structure of hypothetical double perovskite **In/Bi** was obtained by replacing Ag with Bi in the experimental **Ag/In** and relaxing the new geometry.

For the ground-state calculations, we used a plane wave cutoff energy of 60 Ry and sampled the Brillouin zone using a $10 \times 10 \times 10$ uniform **k**-point mesh.

Using the PBE eigensystem as a starting point, we computed the quasiparticle (QP) eigenvalues of the studied perovskites with a one-shot $G_0W_0$ approximation as implemented in the BerkeleyGW software package,[4] with the generalized plasmon-pole method of Godby and Needs.[10] We include fully relativistic spin-orbit coupling (SOC) in the construction of the zeroth-order one-particle Green's function $G_0$ and screened Coulomb interaction $W_0$ for **Ag/Bi**, **In/Bi**, **Ag/Sb**, **K/Bi**, **Na/Bi** and **Pb**. To compute the dielectric screening of **In/Bi** and the **Pb** perovskites we used a half-shifted **k**-point mesh of $6 \times 6 \times 6$ points. For all the other studied double perovskites we used an unshifted Γ-centered $4 \times 4 \times 4$ **k**-point mesh. A polarizability cutoff of 8 Ry and a total of 1400 bands were used for **In/Bi**, while for **Pb** we employed a polarizability cutoff of 15 Ry and a total of 1000 bands, respectively. For all other materials the dielectric screening was computed using a polarizability cutoff of 8 Ry, an energy cutoff of 48 Ry for the bare Coulomb interaction and 600 bands. With the above mentioned computational settings (also shown in Table S1) our QP band gaps are converged to within 0.1 eV. Figures S1 and S2 show the convergence of the QP band gap and static dielectric constant $\varepsilon_\infty$ for **Ag/Bi**, **Ag/In**, **In/Bi** and **Pb** perovskites. For the other halide double perovskites we used the settings that ensured convergence of their

isoelectronic counterparts.

Note that the QP band gaps reported in Table S3 are underestimating experimentally reported (optical) band gaps by ∼0.3 - 0.7 eV.[11] This is primarily due to the well-known starting-point dependence of $G_0W_0$ which is significant for the electronically diverse family of perovskites[11,12] and also thermal fluctuations for $CsPbCl_3$.[13] The agreement can be improved by employing hybrid-functional starting points[14,15] or self-consistent schemes in the calculation of the QP energies.[13] We do not attempt to obtain band gaps in perfect agreement with experiment here, since it would dramatically increase the computational effort without leading to significantly different conclusions.

The PBE and $G_0W_0$@PBE band structures showed in Figure S8 are obtained by Wannier interpolation using the WANNIER90 code.[16] We interpolated both valence and conduction states simultaneously in order to obtain accurate effective masses.

We computed the effective mass tensor by calculating the second derivatives, using finite differences, of the valence and conduction band edges with respect to the **k** vector along three crystallographic directions:

$$\frac{1}{m^*_{\alpha\beta}} = \frac{1}{\hbar^2}\frac{\partial^2 \varepsilon}{\partial k_\alpha \partial k_\beta}, \tag{S2}$$

with $\alpha, \beta = x, y, z$. We obtained the isotropic electron and hole effective masses by diagonalizing the effective mass tensor and computing the harmonic mean:

$$m^* = \frac{3m_1m_2m_3}{m_1m_2 + m_2m_3 + m_3m_1}, \tag{S3}$$

where $m_3 = m_\parallel$ corresponds to the longitudinal direction, while $m_1$ and $m_2$ are the transverse effective masses (along the two perpendicular directions). Furthermore, we computed the reduced effective mass using the mean:

$$\frac{1}{\mu} = \frac{1}{m^*_h} + \frac{1}{m^*_e}, \tag{S4}$$

and quantified the anisotropy of the effective masses using the anisotropy factor:[17]

$$\lambda = \left( \frac{m_{\perp}}{m_{\parallel}} \right)^{1/3}, \tag{S5}$$

where $m_{\perp} = \dfrac{2m_1 m_2}{m_1 + m_2}$ is the harmonic mean of the transverse effective masses and $m_{\parallel} = m_3$ is the longitudinal effective mass.

For **Ag/Bi**, we found that both electron and hole effective masses are highly anisotropic, featuring large differences in the longitudinal component as compared to the transverse ones. Furthermore, we note that for **Ag/In** the hole effective mass is very sensitive to the inclusion of the flat valence band, therefore we computed the hole effective mass by averaging over both the heavy and the light hole.

In Figure S9, we show the QP band structure of the studied perovskites, with the orbital character of the bands represented in colored dots with the size proportional to the percentage contribution of the orbital character to the electronic bands. We note that Cs-derived orbitals do not contribute to the states near the band edges and Cl $s$ and $p$ contributions were omitted for clarity. In line with our previous calculations,[18] we found that **Ag/Bi** features an indirect band gap with the valence band maximum (VBM) at X and the conduction band minimum at Γ. This feature is contrasting with previous calculations[19] that found the indirect band gap to lie between VBM at X and CBM at L. We attribute this shift in the CBM from L to Γ to differences in the crystal structure and pseudopotentials. The VBM is primarily derived from Cl $p$, Ag $d$ and Bi $s$ orbitals, while the CBM is derived from Cl $p$ and Bi $p$ orbitals, in agreement with previous reports.[19,20] In **Ag/In**, the VBM and CBM are at the Γ point due to the contributions of unoccupied In and Ag $s$ orbitals to the CBM (see Figure S9 b)).[21,22] The VBM of the hypothetical direct gap double perovskite **In/Bi** and the isoelectronic single perovskite **Pb** are derived from Cl $p$ and metal $s$ orbitals; their CBM originate from Cl $p$ and metal $p$ orbitals and are strongly affected by SOC (see Figure S9 (c) and (d)).

Table S3 contains a summary of the electronic structure of the studied halide perovskites.

## 1.2 Optical properties

The excitonic properties were computed by solving the Bethe-Salpeter equation (BSE)[23–28] with the Tamm-Dancoff approximation (TDA):[28]

$$(E_{c\boldsymbol{k}}^{QP} - E_{v\boldsymbol{k}}^{QP})A_{vc\boldsymbol{k}}^{S} + \sum_{v'c'\boldsymbol{k'}} \langle vc\boldsymbol{k}| K^{eh} |v'c'\boldsymbol{k'}\rangle A_{v'c'\boldsymbol{k'}}^{S} = \Omega^{S} A_{vc\boldsymbol{k}}^{S}, \tag{S6}$$

where $A_{vc\boldsymbol{k}}^{S}$ are the coefficients of the exciton wave function written in the free electron and hole basis $|vc\boldsymbol{k}\rangle$, $\Omega^{S}$ is the excitation energy, and $K^{eh}$ is the electron-hole interaction kernel.[26]

We calculated the absorption spectra reported in Figure S7 from the imaginary part of the transverse dielectric function:

$$\varepsilon_2(\omega) = \frac{16\pi^2 e^2}{\omega^2} \sum_{S} \left| \hat{e} \cdot \sum_{vc\boldsymbol{k}} A_{vc\boldsymbol{k}}^{S} \langle v\boldsymbol{k}| \hat{p} |c\boldsymbol{k}\rangle \right|^2 \delta(\omega - \Omega^{S}), \tag{S7}$$

where $\hat{p}$ is the momentum operator and $\hat{e}$ is the direction of polarization of light.

For **In/Bi** we constructed the electron-hole interaction kernel $K^{eh}$ on a $6 \times 6 \times 6$ **k**-point grid, using a set of 8 valence and 8 conduction bands, while for the **Pb** single perovskite we calculated $K_{eh}$ using 10 valence and 10 conduction bands. For all other double perovskites $K_{eh}$ is constructed on a $4 \times 4 \times 4$ **k**-point mesh, using 22 valence and 22 conduction bands. To obtain the absorption spectra showed in Figure S7 we interpolated the electron-hole kernel on a fine **k**-point grid of $20 \times 20 \times 20$ points for **Pb**, $30 \times 30 \times 30$ points for **Ag/In** and **In/Bi**, and $10 \times 10 \times 10$ points for all other materials. For the interpolation fine grid we used 4 valence and 2 conduction bands for **Ag/Bi**, 2 valence bands and 1 conduction band for **Ag/In**, 2 valence and 2 conduction bands for **In/Bi** and **Pb**, respectively. All the absorption spectra were obtained using a constant arbitrary Gaussian smearing of 50 meV.

The exciton binding energies reported in Table 1 of the main text (for the first optically (in)active exciton) were defined as the difference between the lowest-energy direct transition and the computed excitation energy of the first (dark) bright excited state.

In all materials studied, the lowest excited state is dipole forbidden. For **Ag/In** and **Na/In**, in which SOC does not affect the valence and conduction band edges, this is a result of the angular-momentum symmetry of the atomic orbitals contributing to the valence and conduction band edges.[29] For the other six materials, the CBM is originating from Bi, Sb or Pb $p$ orbitals, and the inclusion of SOC leads to a splitting of the four degenerate excited states arising from VBM to CBM transitions into a lowest-energy dark and three (nearly) degenerate bright states with oscillator strengths consistent with selection rules.[30–32]

The exciton binding energy of halide perovskites featuring excitons localized in reciprocal space are very sensitive to the density of points in the fine **k**-point grid and notoriously hard to converge.[33–35] Since BSE calculations using very dense fine grids are prohibitive, we employ a patched sampling technique[24,36] that allows us to reach fine grids containing up to $100 \times 100 \times 100$ **k**-points. By using this sampling scheme we take into account only the **k**-points within a small radius around the high symmetry point where the lowest-energy direct transition is taking place. After extensive convergence testing we computed the exciton binding energies using $50 \times 50 \times 50$ **k**-point grid, with patch size of $0.4\,\text{Å}^{-1}$ for **Ag/In**, $100 \times 100 \times 100$ **k**-point grid, with patch size of $0.1\,\text{Å}^{-1}$ for **In/Bi**, $60 \times 60 \times 60$ **k**-point grid, with patch size of $0.3\,\text{Å}^{-1}$ for **Pb** and a $10 \times 10 \times 10$ full **k**-point grid for all the other double perovskites. Our convergence tests with respect to the density of the fine **k**-point grids are presented in Figure S3. For clarity reasons, we summarize the computational settings mentioned above in Table S2.

# 2 Wannier-Mott model

For a standard semiconductor, with parabolic band edges and isotropic dielectric constant, the binding energy of the first excited state (in eV) can be estimated using the standard

Wannier-Mott (or hydrogenic) model:[37]

$$E_{\mathrm{WM}} = \frac{\mu}{{\varepsilon_\infty}^2} \cdot R_H, \tag{S8}$$

where $\mu$ is the reduced effective mass, $\varepsilon_\infty$ is the dielectric constant and $R_H$ represents the Rydberg constant. In Table S4 we report the binding energy of the first (dark) excited state of all studied materials as computed with $G_0W_0$+BSE ($E_{\mathrm{BSE}}$) and compare it with the binding energy calculated from the Wannier-Mott model ($E_{\mathrm{WM}}$) using eq. S8, where the reduced effective mass and dielectric constant are obtained from our $G_0W_0$ calculations. We find that the Wannier-Mott model severely underestimates the binding energy of materials with *non-hydrogenic* excitons.

Following the approach introduced in Ref. 17, we correct the hydrogenic model by taking into account the effect of effective mass anisotropy. To this end we use a modified expression to calculate the Wannier-Mott binding energy:

$$E_{\lambda\mathrm{WM}} = -3 \left(\frac{1}{\varepsilon_\infty}\right)^2 \left(\frac{2}{m_\perp} + \frac{1}{\lambda^2 m_\parallel}\right)^{-1} \left(\frac{\operatorname{arcsinh}\sqrt{\lambda^2-1}}{\sqrt{\lambda^2-1}}\right)^2 R_H, \tag{S9}$$

where $m_\perp$, $m_\parallel$ are the transverse and longitudinal effective masses, respectively and $\lambda$ is the anisotropy factor as defined above. We report the corrected binding energies $E_{\lambda\mathrm{WM}}$ in Table S4 and show that the discrepancy between $E_{\mathrm{WM}}$ and $E_{\mathrm{BSE}}$ can be adjusted (to some extent) by explicitly including the effective mass anisotropy into the model. However, Table S4 shows that this correction is not sufficient to reconcile the first principles results with the hydrogenic model.

We note that calculating the Wannier-Mott binding energy based on either eq. S8 or eq. S9 includes systematic errors, e.g. the step used for calculation of the effective mass via finite differences. In order to avoid these numerical errors, we quantify the agreement with the Wannier-Mott model by probing how the excitonic fine structure in the studied materials compares with the fine structure of an hydrogenic exciton. To this end, we define the energy

difference $E^{\text{fs, WM}} = \frac{4}{3} \cdot (E_{2s} - E_{1s})$, where $E_{1s}$ and $E_{2s}$ are the excitation energies of $1s$ and $2s$ states, respectively, obtained exclusively from our $G_0W_0$+BSE results. The exciton fine structure of materials in which SOC plays an important role feature one low-energy dark excited state, followed by three degenerate bright and a group of four (nearly) degenerate transitions. We assign the $1s$ excited state to the average energy of the first group of (three degenerate) bright transitions and the $2s$ state to the next group of transitions (as described above). We base our assignment on the procedure described in Ref. 41 for extracting Wannier-Mott exciton binding energy from experimental optical absorption spectra. For materials in which SOC does not affect the band edges, i.e. **Ag/In** and **Na/In**, we assign the $1s$ and $2s$ excited states to the first and second group of degenerate transitions, respectively. We note that the excited states in those two groups are optically inactive (as discussed in the main text) and their number depends on the degeneracy of VBM and CBM at the high symmetry point where the lowest-energy direct transition takes place. In Figure S4 we show the $1s$ and $2s$ excited states for each material. Unless otherwise noted, we investigate the nature of excitons in the studied materials by comparing the first principles exciton binding energy $E_{\text{BSE}}$ with the energy difference $E^{\text{fs, WM}}$.

# 3 Dielectric screening

In order to probe the uniformity and isotropy of the dielectric screening, we compute linear absorption spectra (i.e. the imaginary part of the dielectric function), by modifying the real part of the dielectric function such that $\text{Re}[\epsilon(\mathbf{r}, \mathbf{r}'; \omega)] = \varepsilon_\infty$. We find that optical absorption spectra change significantly only for those perovskites in which excitons do not display hydrogenic behavior (see Figure S7). Furthermore, Table S5 shows that the decrease in exciton binding energy upon changing the dielectric screening is at least one order of magnitude larger for the halide double perovskites with *non-hydrogenic* excitons.

# References


(1) Giannozzi, P. et al. QUANTUM ESPRESSO: a modular and open-source software project for quantum simulations of materials. *J. Phys. Condens. Matter* **2009**, *21*, 395502.

(2) Giannozzi, P. et al. Advanced capabilities for materials modelling with Quantum ESPRESSO. *J. Phys. Condens. Matter* **2017**, *29*, 465901.

(3) Perdew, J. P.; Burke, K.; Ernzerhof, M. Generalized Gradient Approximation Made Simple. *Phys. Rev. Lett.* **1996**, *77*, 3865–3868.

(4) Deslippe, J.; Samsonidze, G.; Strubbe, D. A.; Jain, M.; Cohen, M. L.; Louie, S. G. BerkeleyGW: A massively parallel computer package for the calculation of the quasiparticle and optical properties of materials and nanostructures. *Comput. Phys. Commun.* **2012**, *183*, 1269–1289.

(5) Louie, S. G.; Hybertsen, M. S. Theory of quasiparticle energies: Band gaps and excitation spectra in solids. *Int. J. Quantum Chem.* **1987**, *32*, 31–44.

(6) Perdew, J. P.; Zunger, A. Self-interaction correction to density-functional approximations for many-electron systems. *Phys. Rev. B* **1981**, *23*, 5048–5079.

(7) Hamann, D. R. Optimized norm-conserving Vanderbilt pseudopotentials. *Phys. Rev. B* **2013**, *88*, 085117.

(8) van Setten, M. J.; Giantomassi, M.; Bousquet, E.; Verstraete, M. J.; Hamann, D. R.; Gonze, X.; Rignanese, G. M. The PseudoDojo: Training and grading a 85 element optimized norm-conserving pseudopotential table. *Comput. Phys. Commun.* **2018**, *226*, 39–54.

(9) Perdew, J. P.; Ruzsinszky, A.; Csonka, G. I.; Vydrov, O. A.; Scuseria, G. E.; Constantin, L. A.; Zhou, X.; Burke, K. Restoring the density-gradient expansion for exchange in solids and surfaces. *Phys. Rev. Lett.* **2008**, *100*, 136406.

(10) Godby, R. W.; Needs, R. J. Metal-Insulator Transition in Kohn-Sham Theory and Quasiparticle Theory. *Phys. Rev. Lett.* **1989**, *62*, 1169–1172.

(11) Leppert, L.; Rangel, T.; Neaton, J. B. Towards predictive band gaps for halide perovskites: Lessons from one-shot and eigenvalue self-consistent GW. *Phys. Rev. Mater.* **2019**, *3*, 103803.

(12) Filip, M. R.; Eperon, G. E.; Snaith, H. J.; Giustino, F. Steric engineering of metal-halide perovskites with tunable optical band gaps. *Nat. Comm.* **2014**, *5*, 5757.

(13) Wiktor, J.; Reshetnyak, I.; Ambrosio, F.; Pasquarello, A. Comprehensive modeling of the band gap and absorption spectrum of $BiVO_4$. *Phys. Rev. Mater.* **2017**, *1*, 022401.

(14) Wing, D.; Ohad, G.; Haber, J. B.; Filip, M. R.; Gant, S. E.; Neaton, J. B.; Kronik, L. Band gaps of crystalline solids from Wannier-localization based optimal tuning of a screened range-separated hybrid functional. *Proc. Nat. Acad. Sci.* **2020**, *118*, e2104556118.

(15) Gant, S. E.; Haber, J. B.; Filip, M. R.; Sagredo, F.; Wing, D.; Ohad, G.; Kronik, L.; Neaton, J. B. Optimally Tuned Starting Point for Single-Shot *GW* Calculations of Solids. *Phys. Rev. Mater.* **2022**, *6*, 053802.

(16) Pizzi, G. et al. Wannier90 as a community code: new features and applications. *J. Phys. Condens. Matter* **2020**, *32*, 165902.

(17) Schindlmayr, A. Excitons with anisotropic effective mass. *Euro. J. Phys.* **1997**, *18*, 374–376.

(18) Biega, R. I.; Filip, M. R.; Leppert, L.; Neaton, J. B. Chemically Localized Resonant Excitons in Silver-Pnictogen Halide Double Perovskites. *J. Phys. Chem. Lett.* **2021**, *12*, 2057–2063.

(19) Filip, M. R.; Hillman, S.; Haghighirad, A.-A.; Snaith, H. J.; Giustino, F. Band Gaps of the Lead-Free Halide Double Perovskites $Cs_2BiAgCl_6$ and $Cs_2BiAgBr_6$ from Theory and Experiment. *J. Phys. Chem. Lett.* **2016**, *7*, 2579–2585.

(20) McClure, E. T.; Ball, M. R.; Windl, W.; Woodward, P. M. $Cs_2AgBiX_6$ (X = Br, Cl) — New visible light absorbing, lead-free halide perovskite semiconductors. *Chem. Mater.* **2016**, *6*, 1348–1354.

(21) Volonakis, G.; Haghighirad, A. A.; Milot, R. L.; Sio, W. H.; Filip, M. R.; Wenger, B.; Johnston, M. B.; Herz, L. M.; Snaith, H. J.; Giustino, F. $Cs_2InAgCl_6$: A New Lead-free Halide Double Perovskite with Direct Band Gap. *J. Phys. Chem. Lett.* **2017**, *8*, 772–778.

(22) Slavney, A. H.; Connor, B. A.; Leppert, L.; Karunadasa, H. I. A pencil-and-paper method for elucidating halide double perovskite band structures. *Chem. Sci.* **2019**, *10*, 11041.

(23) Hedin, L. New Method for Calculating the One-Particle Green's Function with Application to the Electron-Gas Problem. *Phys. Rev.* **1965**, *139*, A796.

(24) Rohlfing, M.; Louie, S. G. Electron-hole excitations in semiconductors and insulators. *Phys. Rev. Lett.* **1998**, *81*, 2312–2315.

(25) Albrecht, S.; Reining, L.; Del Sole, R.; Onida, G. Ab initio calculation of excitonic effects in the optical spectra of semiconductors. *Phys. Rev. Lett.* **1998**, *80*, 4510.

(26) Rohlfing, M.; Louie, S. G. Electron-hole excitations and optical spectra from first principles. *Phys. Rev. B* **2000**, *62*, 4927.

(27) Onida, G.; Reining, L.; Rubio, A. Electronic excitations: density-functional versus many-body Green's-function approaches. *Rev. Mod. Phys.* **2002**, *74*, 601.

(28) Kronik, L.; Neaton, J. B. Excited-State Properties of Molecular Solids from First Principles. *Annu. Rev. Phys. Chem.* **2016**, *67*, 587–616.

(29) Meng, W.; Wang, X.; Xiao, Z.; Wang, J.; Mitzi, D. B.; Yan, Y. Parity-Forbidden Transitions and Their Impacts on the Optical Absorption Properties of Lead-Free Metal Halide Perovskites and Double Perovskites. *J. Phys. Chem. Lett.* **2017**, *8*, 2999–3007.

(30) Even, J.; Pedesseau, L.; Katan, C.; Kepenekian, M.; Lauret, J.-S.; Sapori, D.; Deleporte, E. A Solid State Physics Perspective on Hybrid Perovskite Semiconductors. *J. Phys. Chem. C* **2015**, 10161–10177.

(31) Becker, M. A. et al. Bright triplet excitons in caesium lead halide perovskites. *Nature* **2018**, *553*, 189–193.

(32) Biffi, G.; Cho, Y.; Krahne, R.; Berkelbach, T. C. Excitons and Their Fine Structure in Lead Halide Perovskite Nanocrystals from Atomistic GW/BSE Calculations. *The J. Phys. Chem. C* **2023**, *127*, 1891–1898.

(33) Bokdam, M.; Sander, T.; Stroppa, A.; Picozzi, S.; Sarma, D. D.; Franchini, C.; Kresse, G. Role of Polar Phonons in the Photo Excited State of Metal Halide Perovskites. *Sci. Rep.* **2016**, *6*, 28618.

(34) Filip, M. R.; Haber, J. B.; Neaton, J. B. Phonon Screening of Excitons in Semiconductors: Halide Perovskites and beyond. *Phys. Rev. Lett.* **2021**, *127*, 067401.

(35) Chen, Y.; Motti, S. G.; Oliver, R. D. J.; Wright, A. D.; Snaith, H. J.; Johnston, M. B.; Herz, L. M.; Filip, M. R. Optoelectronic Properties of Mixed Iodide–Bromide Perovskites from First-Principles Computational Modeling and Experiment. *J. Phys. Chem. Lett.* **2022**, *13*, 4184–4192.

(36) Da Jornada, F. H.; Qiu, D. Y.; Louie, S. G. Nonuniform sampling schemes of the Brillouin zone for many-electron perturbation-theory calculations in reduced dimensionality. *Phys. Rev. B* **2017**, *95*, 035109.

(37) Wannier, G. H. The Structure of Electronic Excitation Levels in Insulating Crystals. *Phys. Rev.* **1937**, *52*, 191–197.

(38) Cappellini, G.; Sole, R. D.; Bechstedt, F. Model dielectric function for semiconductors. *Phys. Rev. B* **1993**, *47*, 9892–9895.

(39) Cucco, B.; Katan, C.; Even, J.; Kepenekian, M.; Volonakis, G. Fine Structure of Excitons in Vacancy-Ordered Halide Double Perovskites. *ACS Mater. Lett.* **2023**, *5*, 52–59.

(40) Kavanagh, S. R.; Savory, C. N.; Liga, S. M.; Konstantatos, G.; Walsh, A.; Scanlon, D. O. Frenkel Excitons in Vacancy-Ordered Titanium Halide Perovskites ($Cs_2TiX_6$). *The J. Phys. Chem. Lett.* **2022**, *13*, 10965–10975.

(41) Miyata, A.; Mitioglu, A.; Plochocka, P.; Portugall, O.; Wang, J. T.-W.; Stranks, S. D.; Snaith, H. J.; Nicholas, R. J. Direct measurement of the exciton binding energy and effective masses for charge carriers in organic–inorganic tri-halide perovskites. *Nat. Phys.* **2015**, *11*, 582–587.

Table S1: Computational settings for electronic structure calculations.

*hydrogenic*

| **Theory level** | **Input parameter** | **Pb** | **In/Bi** | **Ag/In** | **Na/In** |
|---|---|---|---|---|---|
| | lattice parameter | 5.73 Å | 5.71 Å | 5.17 Å | 5.23 Å |
| DFT | cutoff energy | 70 Ry | 60 Ry | 60 Ry | 60 Ry |
| | **k**-point grid | $8 \times 8 \times 8$ | $10 \times 10 \times 10$ | $10 \times 10 \times 10$ | $10 \times 10 \times 10$ |
| *GW* | cutoff energy for $\varepsilon$ | 15 Ry | 8 Ry | 8 Ry | 8 Ry |
| | cutoff energy for $\Sigma$ | 70 Ry | 60 Ry | 60 Ry | 60 Ry |
| | total number of bands | 1000 | 1400 | 600 | 600 |

*non-hydrogenic*

| **Theory level** | **Input parameter** | **Ag/Bi** | **Ag/Sb** | **Na/Bi** | **K/Bi** |
|---|---|---|---|---|---|
| | lattice parameter | 5.39 Å | 5.33 Å | 5.39 Å | 5.62 Å |
| DFT | cutoff energy | 60 Ry | 60 Ry | 60 Ry | 60 Ry |
| | **k**-point grid | $10 \times 10 \times 10$ | $10 \times 10 \times 10$ | $10 \times 10 \times 10$ | $10 \times 10 \times 10$ |
| *GW* | cutoff energy for $\varepsilon$ | 8 Ry | 10 Ry | 8 Ry | 8 Ry |
| | cutoff energy for $\Sigma$ | 60 Ry | 60 Ry | 60 Ry | 60 Ry |
| | total number of bands | 600 | 600 | 600 | 600 |

Table S2: Computational settings for optical properties calculations.

*hydrogenic*

| **Input parameter** | **Pb** | **In/Bi** | **Ag/In** | **Na/In** |
|---|---|---|---|---|
| states coarse grid | 10 occupied<br>10 unoccupied | 8 occupied<br>8 unoccupied | 22 occupied<br>22 unoccupied | 24 occupied<br>20 unoccupied |
| states fine grid | 2 occupied<br>2 unoccupied | 2 occupied<br>2 unoccupied | 2 occupied<br>1 unoccupied | 3 occupied<br>1 unoccupied |
| coarse grid | $6 \times 6 \times 6$ | $6 \times 6 \times 6$ | $4 \times 4 \times 4$ | $4 \times 4 \times 4$ |
| fine grid absorption spectra | $20 \times 20 \times 20$ | $30 \times 30 \times 30$ | $30 \times 30 \times 30$ | $20 \times 20 \times 20$ |
| smearing | 50 meV | 50 meV | 50 meV | 50 meV |
| fine grid binding energy | $60 \times 60 \times 60$ | $100 \times 100 \times 100$ | $50 \times 50 \times 50$ | $20 \times 20 \times 20$ |
| patch size | $0.25\,\text{Å}^{-1}$ | $0.10\,\text{Å}^{-1}$ | $0.40\,\text{Å}^{-1}$ | — |

*non-hydrogenic*

| **Input parameter** | **Ag/Bi** | **Ag/Sb** | **Na/Bi** | **K/Bi** |
|---|---|---|---|---|
| states coarse grid | 22 occupied<br>22 unoccupied | 22 occupied<br>22 unoccupied | 24 occupied<br>20 unoccupied | 24 occupied<br>20 unoccupied |
| states fine grid | 4 occupied<br>2 unoccupied | 6 occupied<br>6 unoccupied | 6 occupied<br>6 unoccupied | 6 occupied<br>6 unoccupied |
| coarse grid | $4 \times 4 \times 4$ | $4 \times 4 \times 4$ | $4 \times 4 \times 4$ | $4 \times 4 \times 4$ |
| fine grid absorption spectra | $10 \times 10 \times 10$ | $12 \times 12 \times 12$ | $10 \times 10 \times 10$ | $10 \times 10 \times 10$ |
| smearing | 50 meV | 50 meV | 50 meV | 50 meV |
| fine grid binding energy | $10 \times 10 \times 10$ | $12 \times 12 \times 12$ | $10 \times 10 \times 10$ | $10 \times 10 \times 10$ |
| patch size | — | — | — | — |

Table S3: PBE and $G_0W_0$@PBE band gap and lowest-energy direct transition (in eV), effective masses at the band edges (in units of the electron rest mass $m_0$) and effective mass anisotropy.

*hydrogenic*

| System | Level of theory | Band gap (eV) fundamental | lowest direct | Effective masses ($m_0$) $m_h^*$ | $m_e^*$ | $\mu$ | $\lambda_\mu$ |
|---|---|---|---|---|---|---|---|
| **Pb** | PBE | 0.98 | 0.98 | 0.191 | 0.181 | 0.093 | 1.00 |
| | $G_0W_0$ | 2.25 | 2.25 | 0.205 | 0.214 | 0.105 | 1.00 |
| **In/Bi** | PBE | 0.21 | 0.21 | 0.066 | 0.075 | 0.035 | 0.95 |
| | $G_0W_0$ | 0.90 | 0.90 | 0.098 | 0.110 | 0.052 | 0.94 |
| **Ag/In** | PBE | 0.91 | 0.91 | 0.800 | 0.262 | 0.197 | 1.04 |
| | $G_0W_0$ | 2.09 | 2.09 | 0.650 | 0.270 | 0.191 | 1.05 |
| **Na/In** | PBE | 3.09 | 3.09 | 1.473 | 0.454 | 0.347 | 0.96 |
| | $G_0W_0$ | 5.52 | 5.52 | 1.727 | 0.431 | 0.345 | 0.96 |

*non-hydrogenic*

| System | Level of theory | Band gap (eV) fundamental | lowest direct | Effective masses ($m_0$) $m_h^*$ | $m_e^*$ | $\mu$ | $\lambda_\mu$ |
|---|---|---|---|---|---|---|---|
| **Ag/Bi** | PBE | 1.37 | 1.83 | 0.367 | — | 0.367 | 1.31 |
| | $G_0W_0$ | 2.15 | 2.64 | 0.296 | 1.305 | 0.241 | 1.29 |
| **Ag/Sb** | PBE | 1.24 | 2.30 | 0.399 | — | 0.399 | 1.29 |
| | $G_0W_0$ | 2.13 | 3.20 | 0.315 | — | 0.315 | 1.23 |
| **Na/Bi** | PBE | 2.94 | 3.15 | 2.295 | 1.188 | 0.783 | 0.82 |
| | $G_0W_0$ | 4.71 | 4.93 | 1.479 | 1.370 | 0.711 | 0.86 |
| **K/Bi** | PBE | 3.06 | 3.06 | 0.728 | — | 0.728 | 0.83 |
| | $G_0W_0$ | 4.99 | 4.99 | 0.586 | — | 0.586 | 0.88 |

Table S4: Exciton binding energy (in eV) of the first (dark) excited state as computed with $G_0W_0$+BSE approach ($E_{\text{BSE}}$), with standard[37] (hydrogenic) ($E_{\text{WM}}$ from eq. S8) and corrected[17] ($E_{\lambda\text{WM}}$ from eq. S9) Wannier-Mott model. For clarity reasons the absolute deviations (in meV) of the two models with respect to the first principles results are reported as $\Delta = |E_{\text{BSE}} - E_{\text{WM}}|$ and $\Delta_\lambda = |E_{\text{BSE}} - E_{\lambda\text{WM}}|$.

*hydrogenic*

| **System** | | **Wannier-Mott**[37] | | **Schindlmayr**[17] | |
|---|---|---|---|---|---|
| | $E_{\mathbf{BSE}}$ **(eV)** | $E_{\mathbf{WM}}$ **(eV)** | $\Delta$ **(meV)** | $E_{\lambda\mathbf{WM}}$ **(eV)** | $\Delta_\lambda$ **(meV)** |
| **Pb** | 0.105 | 0.106 | 0.65 | 0.105 | 0.45 |
| **In/Bi** | 0.021 | 0.022 | 0.01 | 0.021 | 0.04 |
| **Ag/In** | 0.176 | 0.185 | 8.56 | 0.181 | 4.63 |
| **Na/In** | 0.642 | 0.609 | 33.30 | 0.623 | 18.98 |

*non-hydrogenic*

| **System** | | **Wannier-Mott**[37] | | **Schindlmayr**[17] | |
|---|---|---|---|---|---|
| | $E_{\mathbf{BSE}}$ **(eV)** | $E_{\mathbf{WM}}$ **(eV)** | $\Delta$ **(meV)** | $E_{\lambda\mathbf{WM}}$ **(eV)** | $\Delta_\lambda$ **(meV)** |
| **Ag/Bi** | 0.426 | 0.163 | 263.55 | 0.160 | 266.02 |
| **Ag/Sb** | 0.639 | 0.200 | 438.91 | 0.316 | 322.78 |
| **Na/Bi** | 1.953 | 1.013 | 939.63 | 1.230 | 722.95 |
| **K/Bi** | 2.091 | 1.069 | 1021.79 | 1.113 | 977.26 |

Table S5: Exciton binding energy (in eV) of the first (dark) excited state as computed with $G_0W_0$+BSE approach using the full dielectric matrix ($E_{\mathrm{BSE}}$) and with the modified uniform dielectric screening ($E_{\varepsilon_\infty}$), and their difference $\Delta_{\varepsilon_\infty} = E_{\mathrm{BSE}} - E_{\varepsilon_\infty}$.

*hydrogenic*

| **System** | $E_{\mathbf{BSE}}$ **(eV)** | $E_{\varepsilon_\infty}$ **(eV)** | $\Delta_{\varepsilon_\infty}$ **(meV)** |
|---|---|---|---|
| **Pb** | 0.105 | 0.101 | 4.16 |
| **In/Bi** | 0.021 | 0.016 | 5.12 |
| **Ag/In** | 0.176 | 0.169 | 10.89 |
| **Na/In** | 0.642 | 0.599 | 38.67 |

*non-hydrogenic*

| **System** | $E_{\mathbf{BSE}}$ **(eV)** | $E_{\varepsilon_\infty}$ **(eV)** | $\Delta_{\varepsilon_\infty}$ **(meV)** |
|---|---|---|---|
| **Ag/Bi** | 0.426 | 0.305 | 101.26 |
| **Ag/Sb** | 0.639 | 0.516 | 123.77 |
| **Na/Bi** | 1.953 | 1.586 | 364.97 |
| **K/Bi** | 2.091 | 1.714 | 375.51 |

Table S6: The RPA dielectric constant $\varepsilon_\infty$ and the fitting parameters ($\alpha$ and Thomas-Fermi wave vector $q_{TF}$) used for the model dielectric functions[38] represented in Figure 3(a) of the main text and Figure S10.

| | **Pb** | **In/Bi** | **Ag/In** | **Na/In** | **Ag/Bi** | **Ag/Sb** | **Na/Bi** | **K/Bi** |
|---|---|---|---|---|---|---|---|---|
| $\varepsilon_\infty$ | 3.67 | 5.63 | 3.75 | 2.78 | 4.49 | 4.63 | 3.09 | 2.73 |
| $\alpha$ | 2.562 | 1.926 | 2.684 | 1.135 | 2.372 | 2.160 | 1.213 | 1.333 |
| $q_{TF}$ | 0.891 | 0.607 | 1.107 | 0.859 | 0.882 | 0.870 | 0.818 | 0.812 |

Table S7: DFT-PBE band gap and lowest-energy direct transition (in eV), effective masses at the band edges (in units of the electron rest mass $m_0$) and effective mass anisotropy of vacancy-ordered double perovskites discussed in the main text, with the crystal structures as reported in Refs. 39 and 40.

| **System** | **Band gap (eV)** | | **Effective masses ($m_0$)** | | | |
|---|---|---|---|---|---|---|
| | fundamental | lowest direct | $m_h^*$ | $m_e^*$ | $\mu$ | $\lambda_\mu$ |
| $Cs_2TeBr_6$ | 2.04 $L \to \Gamma$ | 2.19 $L$ | 1.372 | 0.672 | 0.451 | 1.21 |
| $Cs_2TiBr_6$ | 1.22 $X \to \Gamma$ | 1.26 $\Gamma$ | 0.955 | 2.219 | 0.667 | 0.87 |
| $Cs_2SnBr_6$ | 1.28 $\Gamma$ | 1.28 $\Gamma$ | 0.605 | 0.282 | 0.192 | 0.94 |

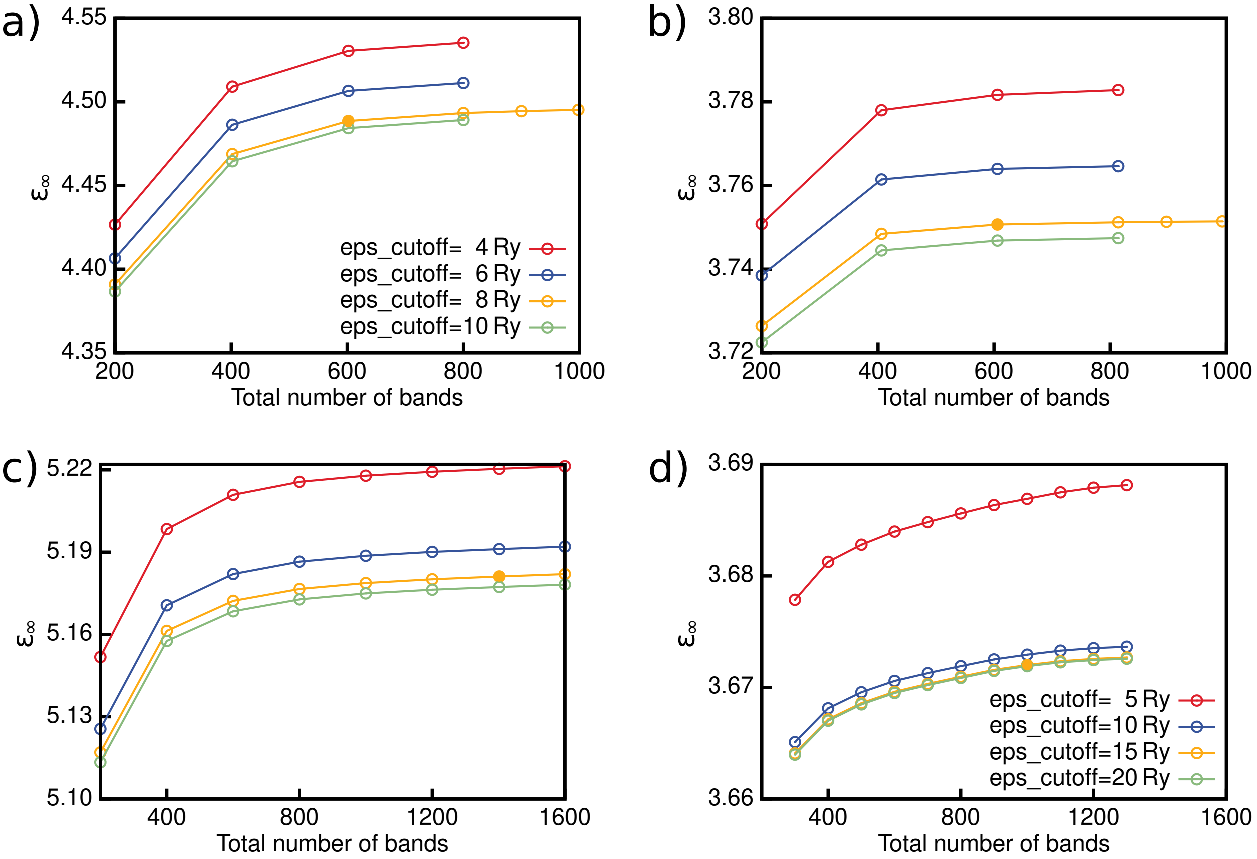


Figure S1: Convergence of the static dielectric constant $\varepsilon_\infty$, as computed within $G_0W_0$, with respect to polarizability cutoff and total number of bands for a) **Ag/Bi**; b) **Ag/In**; c) **In/Bi** and d) **Pb**. The closed symbols show the parameters that ensure convergence.

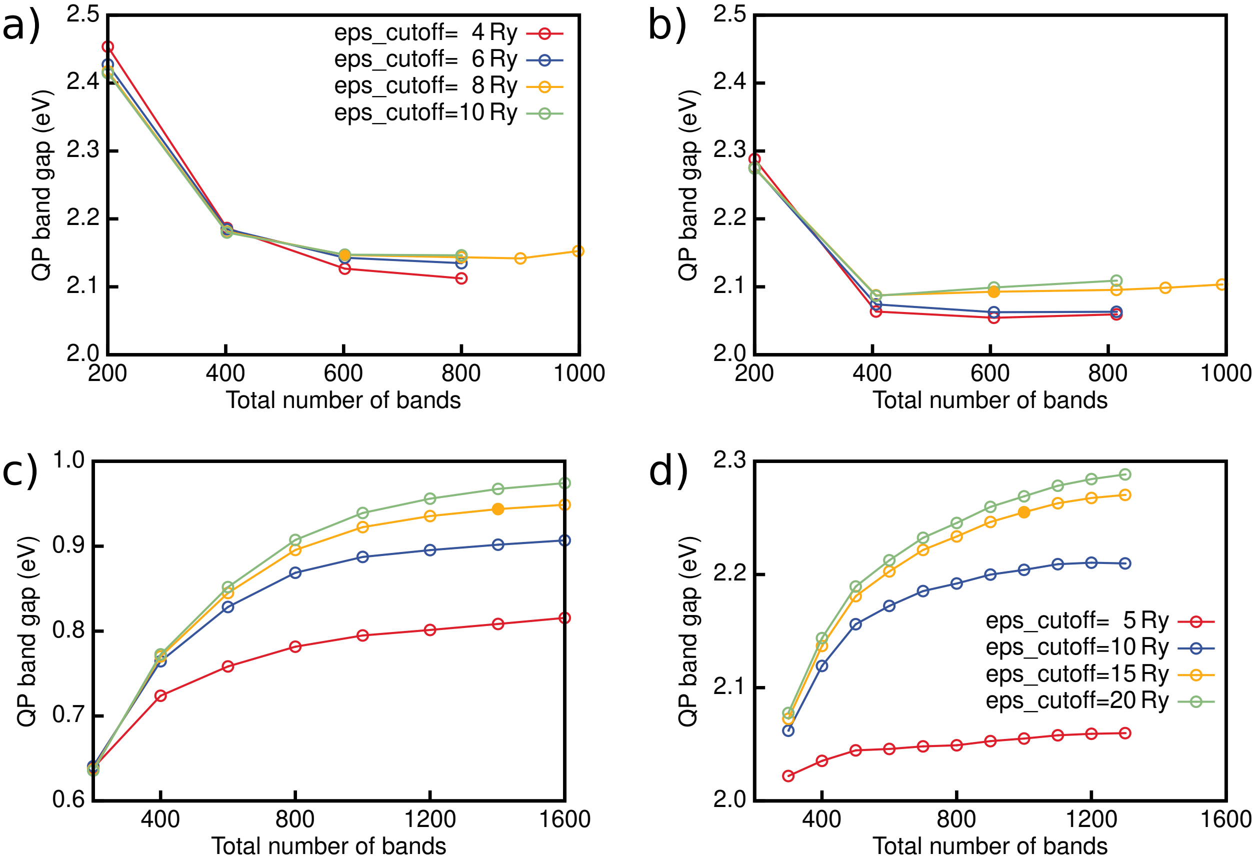


Figure S2: Convergence of QP band gap, as computed within $G_0W_0$, with respect to polarizability cutoff and total number of bands for a) **Ag/Bi**; b) **Ag/In**; c) **In/Bi** and d) **Pb**. The closed symbols show the parameters that ensure convergence.

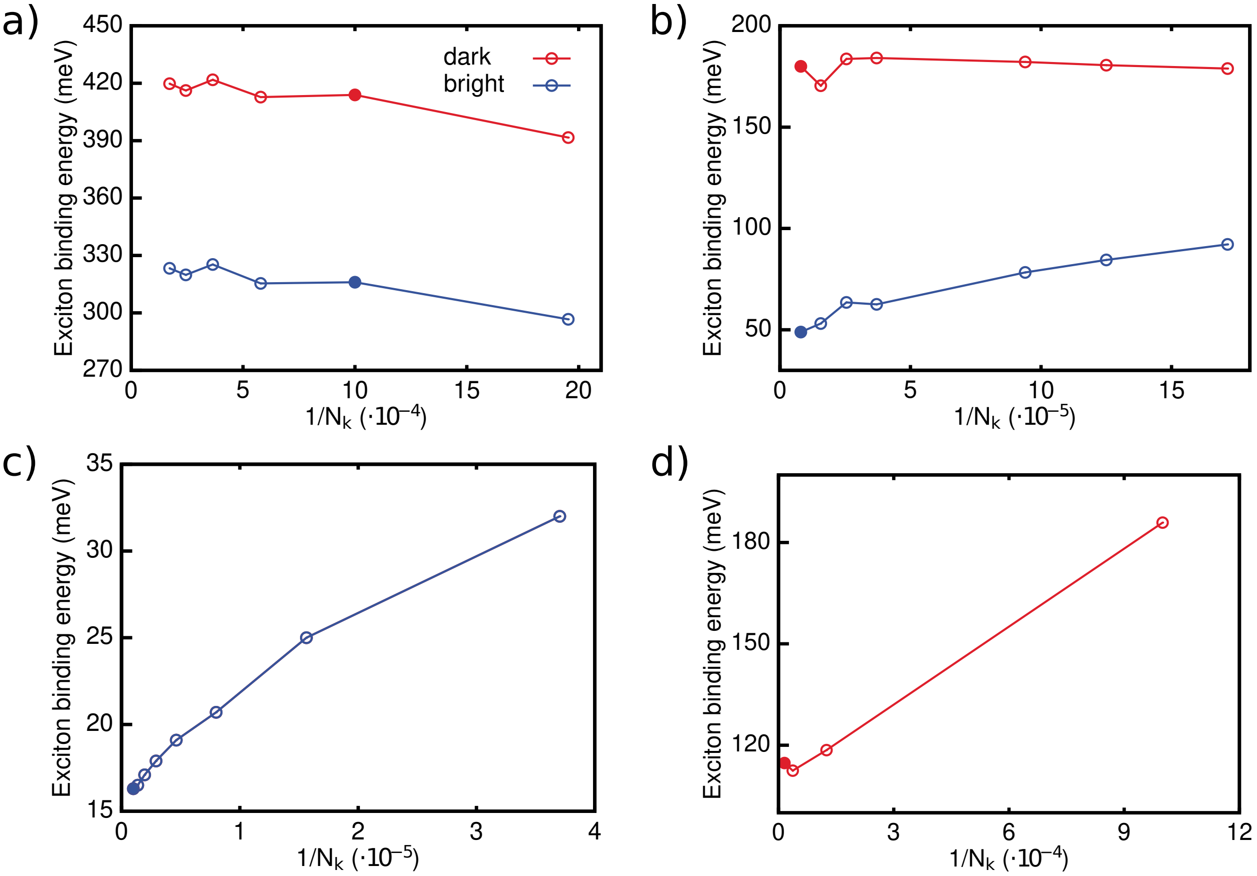


Figure S3: Convergence of exciton binding energy of the first dark (in red)and first bright (in blue) excited states for a) **Ag/Bi**; b) **Ag/In**; c) **In/Bi** and d) **Pb**. The closed symbols show the parameters that ensure convergence.

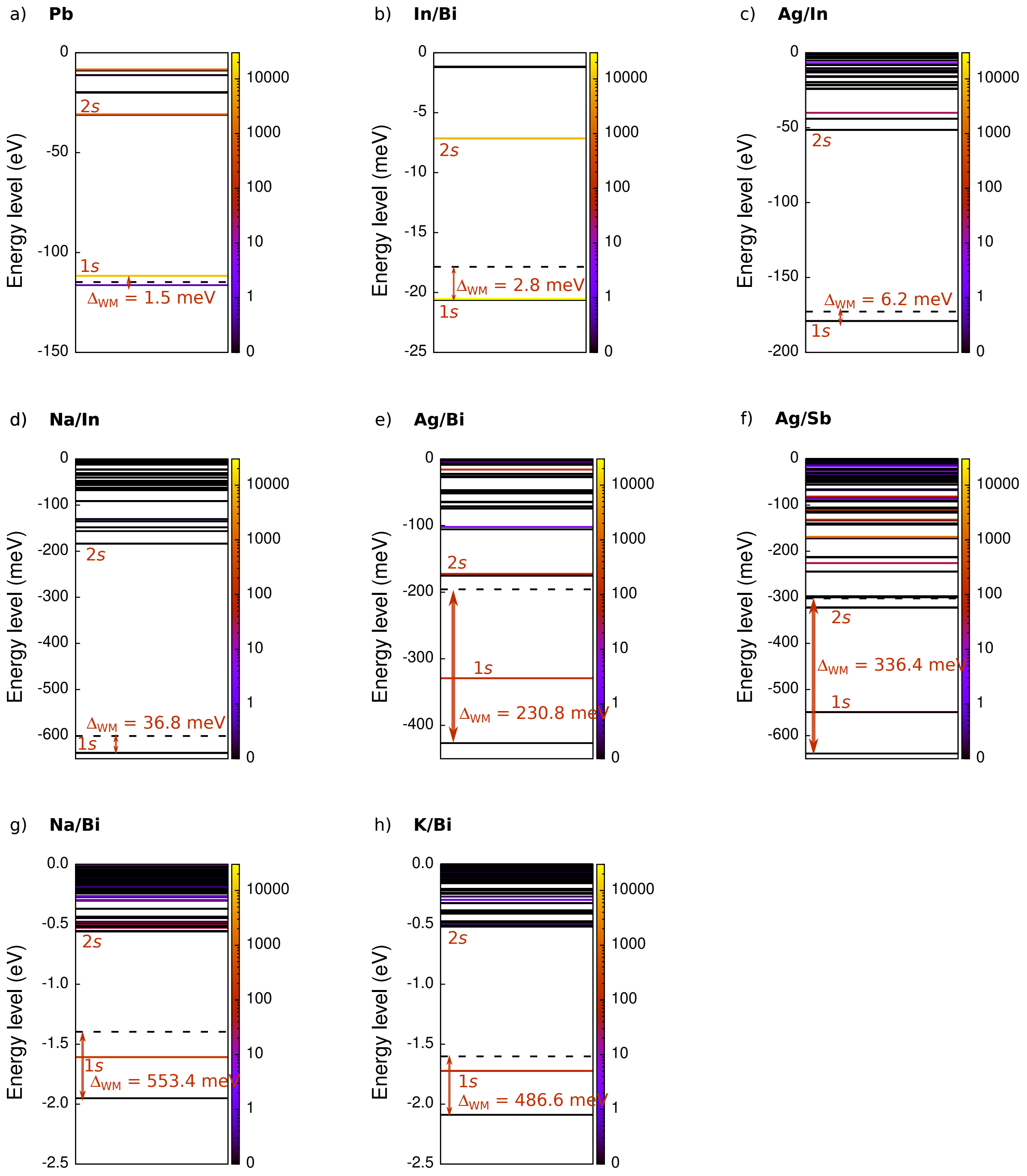


Figure S4: Exciton diagram calculated within the $G_0W_0$+BSE approach for (a) **Pb**, (b) **In/Bi**, (c) **Ag/In**, (d) **Na/In**, (e) **Ag/Bi**, (f) **Ag/Sb**, (g) **Na/Bi** and (h) **K/Bi**, with the $1s$ and $2s$ excited states labeled in red (see text for details on the assignment of these states). The color scale represents the oscillator strength such that black lines correspond to dark transitions. The energy of the $1s$ excitonic state as computed with the hydrogenic Wannier-Mott model is represented by a black dashed line. For each material the absolute deviation from the Wannier-Mott model $\Delta_{\mathrm{WM}}$ is indicated by a red arrow and the corresponding value.

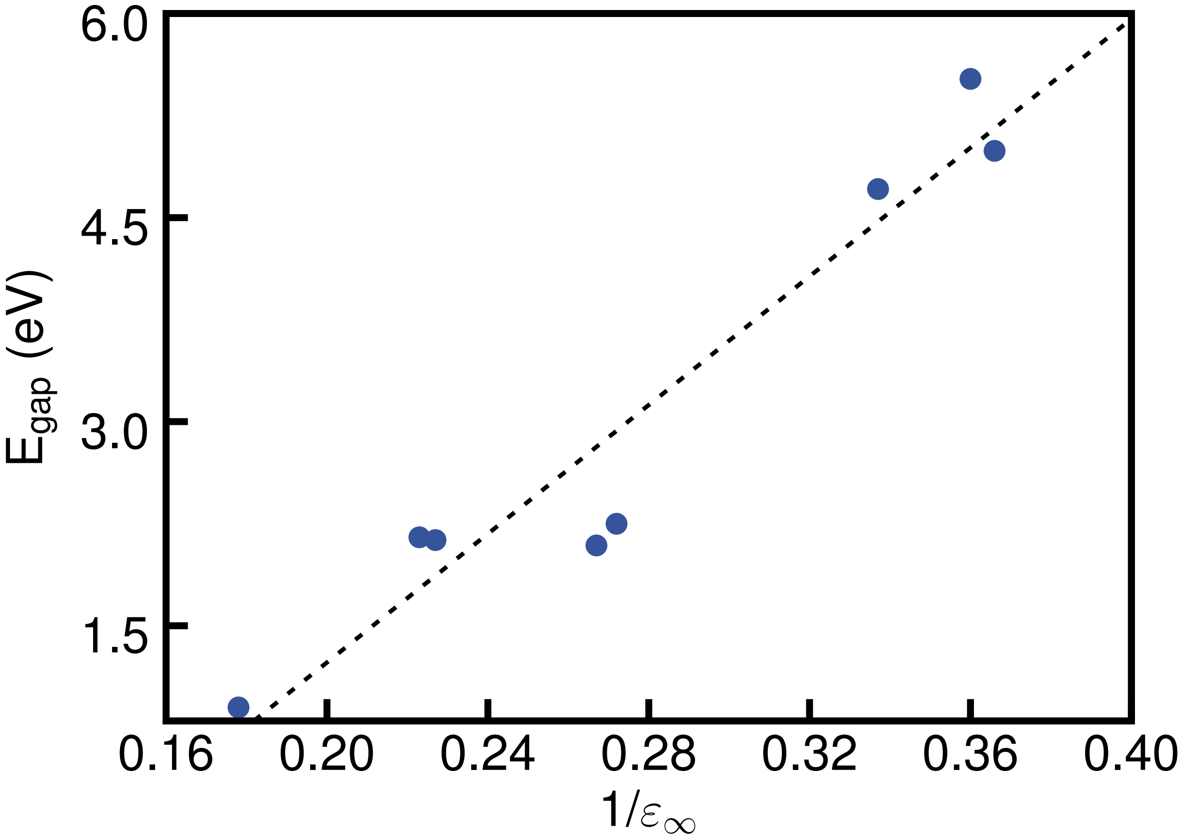


Figure S5: Fundamental QP band gap as computed within the $G_0W_0$ approximation as function of the inverse of static dielectric constant $\varepsilon_\infty$. The dashed line serves as a guide to the eye.

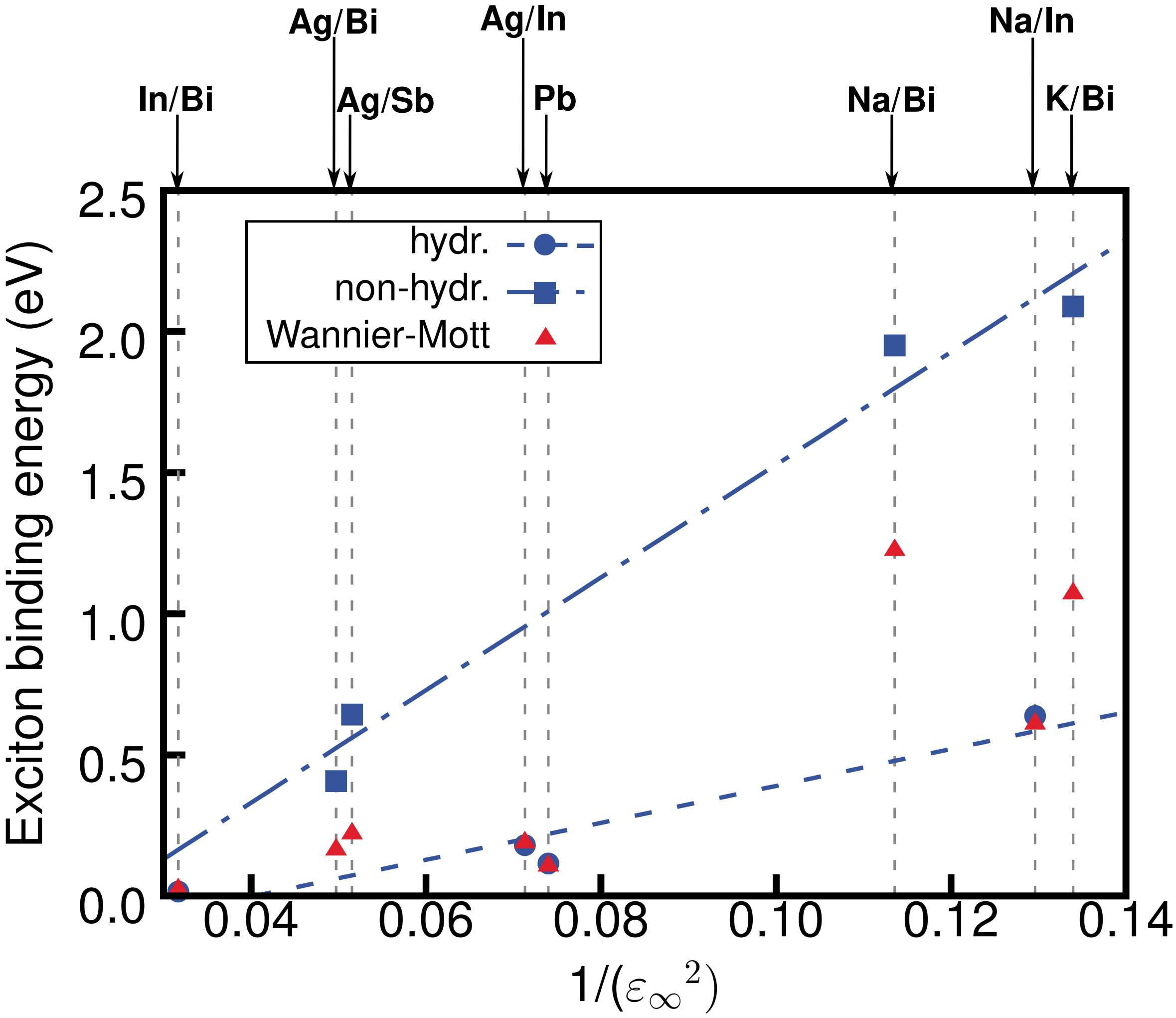


Figure S6: Exciton binding energy (in eV) as computed within the $G_0W_0$+BSE approach (blue) and Wannier-Mott model as defined in eq. S8 (red), as function of the squared inverse of static dielectric constant $\varepsilon_\infty$.

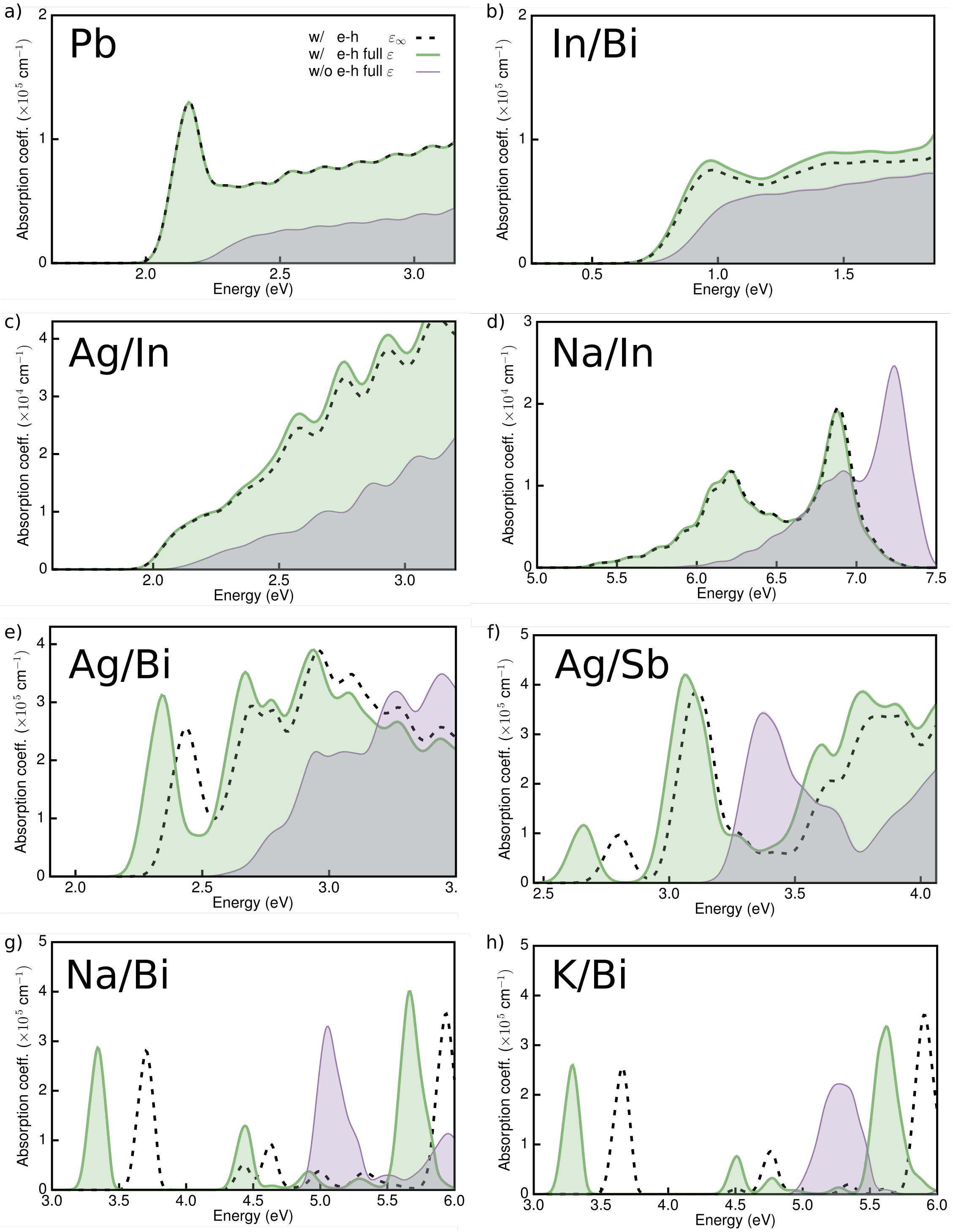


Figure S7: Linear optical absorption spectra, from 0.1 eV below the onset to 1.5 eV above the onset, calculated using the random phase approximation (RPA) (purple), the $G_0W_0$+BSE approach with full dielectric matrix $\epsilon(\mathbf{r}, \mathbf{r}'; \omega)$ (green) and with uniform dielectric matrix $\epsilon(\mathbf{r}, \mathbf{r}'; \omega) = \varepsilon_\infty$ (black dashed line). The absorption coefficient of **Ag/In** and **Na/In** is one order of magnitude lower than that of the other materials.

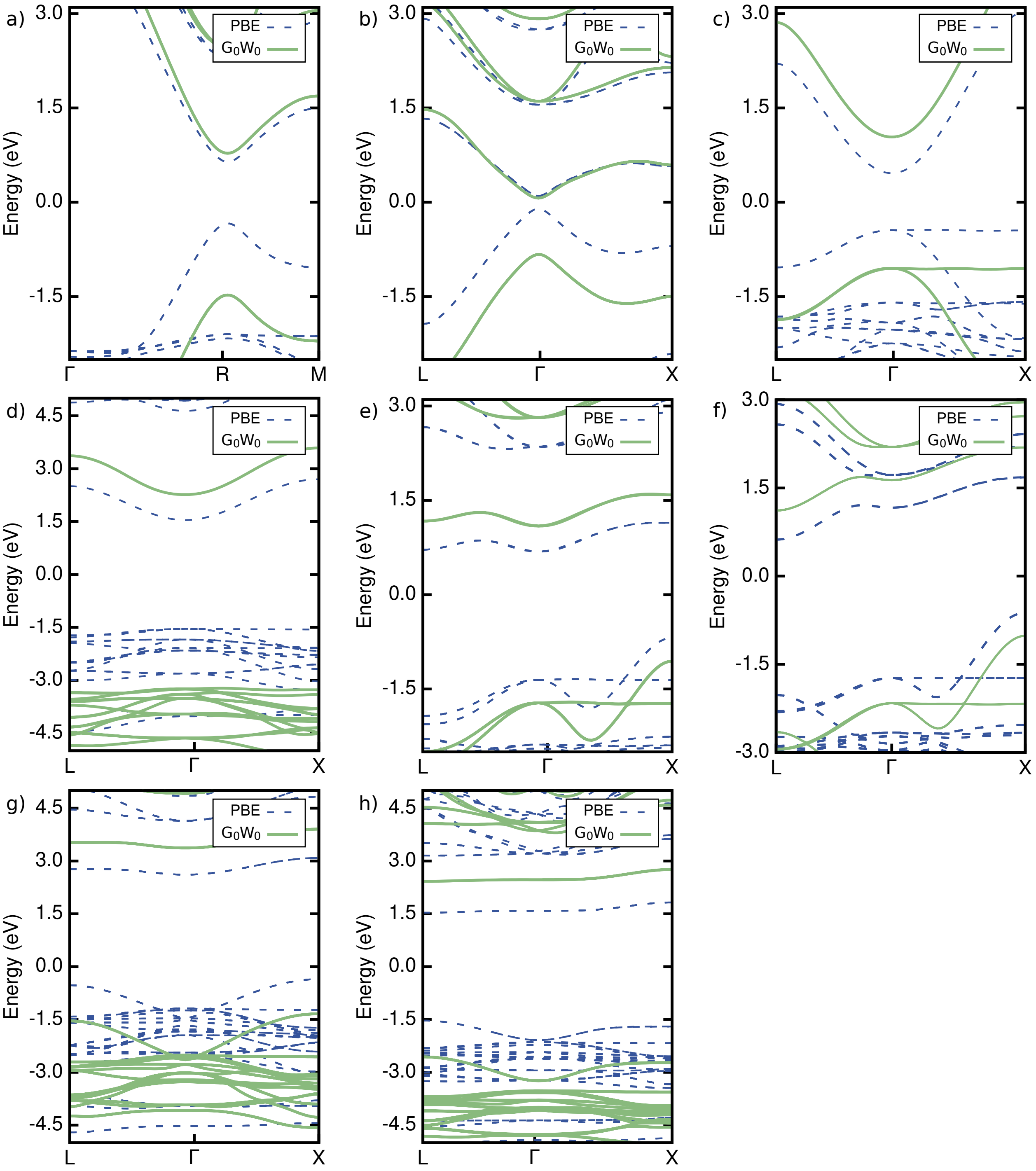


Figure S8: DFT-PBE (blue dashed line) and $G_0W_0$@PBE (green solid line) Wannierized band structures of (a) **Pb**, (b) **In/Bi**, (c) **Ag/In**, (d) **Na/In**, (e) **Ag/Bi**, (f) **Ag/Sb**, (g) **Na/Bi**, (h) **K/Bi**.

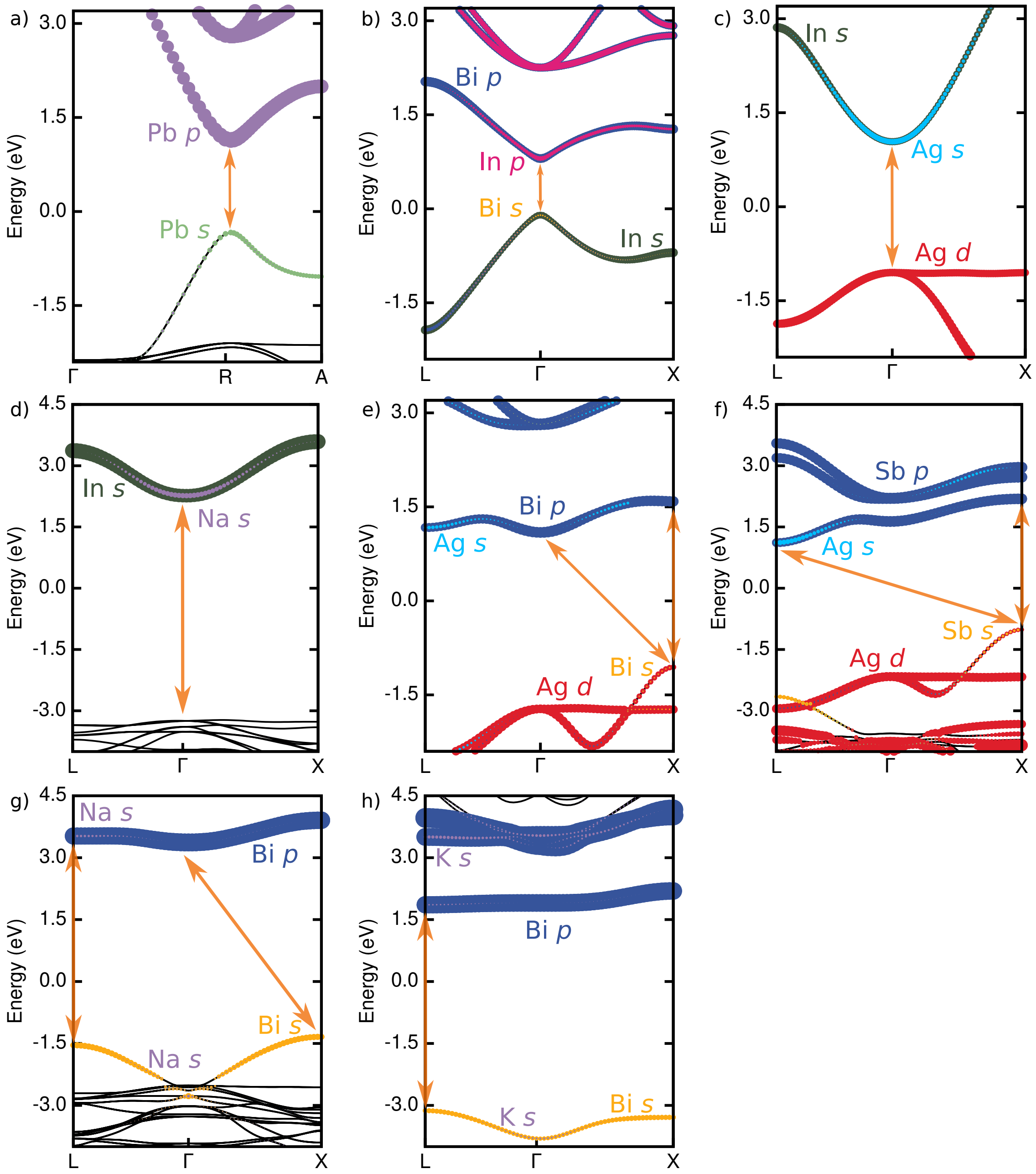


Figure S9: QP band structure along Γ $[0, 0, 0]$ - R $[1/2, 1/2, 1/2]2\pi/a$ - A $[1/2, 1/2, 0]2\pi/a$ for (a) **Pb** and along L $[1/2, 1/2, 1/2]2\pi/a$ - Γ $[0, 0, 0]$ - X $[0, 1, 0]2\pi/a$ for (b) **In/Bi**, (c) **Ag/In**, (d) **Na/In**, (e) **Ag/Bi**, (f) **Ag/Sb**, (g) **Na/Bi** and (h) **K/Bi**. The orbital character of the bands is represented in colored dots with the size proportional to the percentage contribution of the orbital character to the electronic bands. Cs-derived orbitals do not contribute to the states near the band edges and Cl *s* and *p* contributions were omitted for clarity. The arrows mark the fundamental band gap and the lowest direct transition for each material.

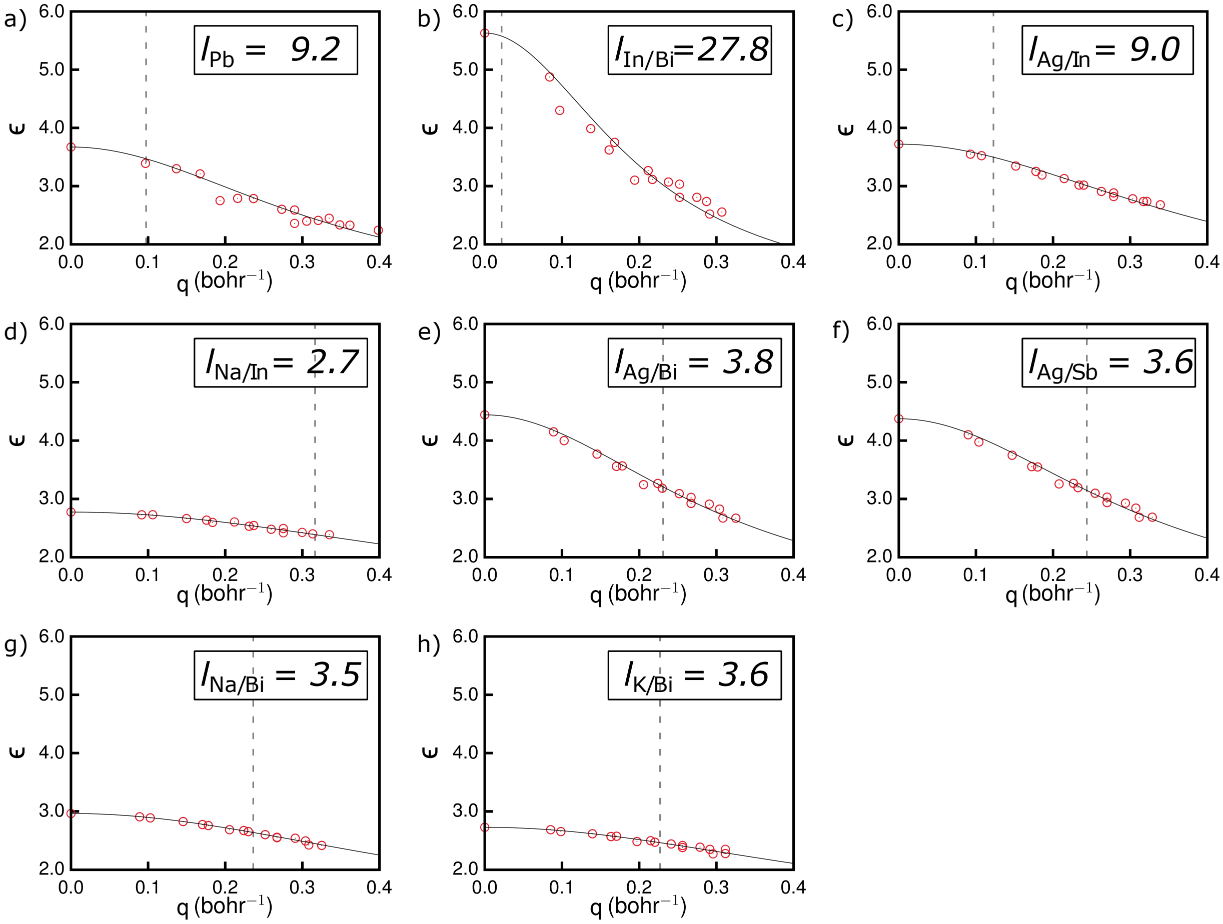


Figure S10: Head of dielectric matrix $\epsilon$ in reciprocal space as computed from $G_0W_0$ (red open circles), fitted with the model dielectric function as described in the text (black solid line), and screening-length parameter $l = \frac{q_{TF}}{k_x}$. The gray dashed line shows the exciton extent in **k**-space defined as the point that includes 99 % of the exciton probability density of the first excited state, as computed within $G_0W_0$+BSE.

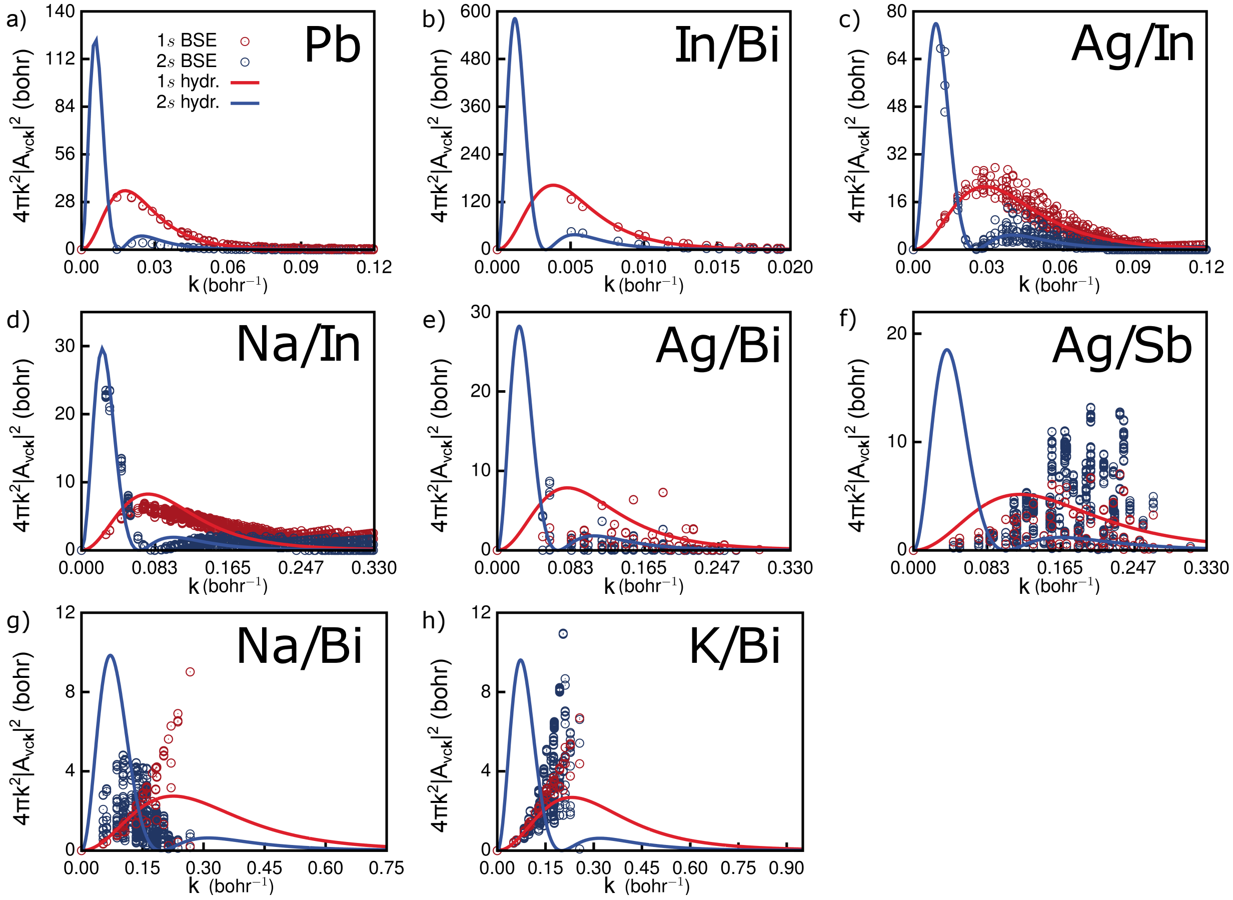


Figure S11: Exciton radial probability density in reciprocal space as computed from $G_0W_0$+BSE (empty disks) and as predicted by the Wannier-Mott model (solid lines) for $1s$ (red) and $2s$ (blue) states.
